# Preliminary Understanding of Abdominal Aortic and Common Iliac Artery Diameters on Abdominal CT in Ethiopian Adults: A Facility-Based Cross-Sectional Study

**DOI:** 10.4314/ejhs.v32i1.4S

**Published:** 2022-10

**Authors:** Fetahi Minichil, Kebede Tesfaye, Henok Zeleke

**Affiliations:** 1 Addis Ababa University, School of Medicine, College of Health Science, Department of Radiology

**Keywords:** Abdominal aorta, Common Iliac Arteries, Abdominal CT, Ethiopia

## Abstract

**Background:**

Mean aortic and common iliac artery diameters are the best indicators for the diagnosis of aortic and iliac ectasia and aneurysm, as well as the appropriate selection of angiographic catheter size and grafts for endovascular procedures. Currently, there is a lack of evidence regarding the normal abdominal aortic and common iliac artery diameters in Ethiopian adults. This study aimed to assess the mean diameter and associated factors of the abdominal aorta and common iliac arteries on abdominal CT scans of Ethiopian adults visiting Tikur Anbessa Specialized Hospital, Addis Ababa, Ethiopia.

**Methods:**

Institution-based prospective cross-sectional study was conducted. A convenience sampling method was employed. Data were collected from consecutive eligible adults who came for abdominal CT scans during the study period, using interviewer-administered structured questionnaires. The data was cleaned and analyzed using SPSS version 22. Student t-test and Pearson correlation were used to perform statistical analysis and the results were presented using tables and figures.

**Results:**

There were a total of 136 study participants of whom eighty-one(59.6%) were females and fifty-five (40.4%) were males. The mean age was 48.5 ± 13 with a range of 23 to77 years. The mean transverse diameter of the aorta at the aortic hiatus (T12)level was 2.30 ± 0.25cm in males and 2.03±0.19cm in females. The mean transverse diameter of the suprarenal aorta was 2.04 ± 0.21cm in males and 1.83 ± 0.21 cm in females while the infrarenal one was 1.77 ± 0.16cm in males and 1.54 ± 0.15cm in females. Participants who are male and older with large body Surface Area were found to have relatively larger aortic and iliac diameters.

**Conclusion:**

In this study, the mean diameter of the aorta and common iliac artery was significantly associated with age, sex, and BSA

## Introduction

The abdominal aorta (AA) is the largest vessel in the abdominal cavity originating at the hiatus of the diaphragm at the level of the twelfth thoracic vertebra. It descends anterior to the lumbar vertebrae to end at the lower border of the 4th lumbar vertebra, slightly to the left of the midline, by dividing into two common iliac arteries (1,2). Its length in adults is approximately 13cm(2). The diameter of the AA and common iliac arteries is important to be evaluated in abdominal computed tomography (CT) scans since it is frequently affected by vascular disorders. These disorders include aortic aneurysm, aortic dissection, atherosclerosis, and connective tissue disorders (3).

The presence of abdominal aortic and iliac artery diseases are mostly reflections of generalized vascular diseases like atherosclerosis. Sometimes Aortic diseases may be localized lesions as in mycotic and traumatic changes. The most common abdominal aortic disease being aneurysm, change in the size of caliber is the basis for establishing the diagnosis even if imaging plays more than determining the aortic diameter.

Because of the retroperitoneal location of the abdominal aorta and overlying bowel gas together with operator dependability of ultrasound examination, MDCT is now a widely used non-invasive technique to investigate the aorta (4). Moreover, a higher degree of precision cannot be made with ultrasound, and ultrasound measurement of the abdominal aorta and iliac arteries may give higher or lower values compared with MDCT evaluation (5, 6). There is also marked interobserver and intra-observer variability with ultrasound measurements (7, 8). Detailed anatomy of the abdominal aorta and its branches can be demonstrated on axial and on thin section reconstructed images at different planes. In addition, the use of 3D and volume rendering applications on advanced CT workstations makes MDCT a popular modality for aortic evaluation (9).

Despite the fact that radiologic evaluation of the AA and common iliac arteries can profoundly help in the diagnosis of these potentially asymptomatic vascular disorders, it will also help the recently introduced vascular surgery and interventional Radiology training by providing a preliminary understanding of our populations' aorto-iliac dimensions in the process of selection of angiographic catheter size for specific endovascular procedures. However, there is a paucity of such studies that evaluate the diameter of AA and common iliac arteries in Ethiopian adults. Thus, this study is aimed to assess the mean diameter of AA and common iliac arteries measured on abdominal CT scan at Tikur Anbessa Specialized Hospital (TASH) and its associations with some clinical parameters such as sex, age, and body mass index (BMI).

## Methods

**Study area**: The study was conducted in TASH, located in Addis Ababa, the capital city of Ethiopia. It is the largest and oldest public hospital in the country providing a high level of clinical care for millions of people and training to health science students from different parts of the country and the Horn of Africa. The hospital is selected for this study because it serves a relatively large size of population from different parts of the country with a range of radiologic facilities. The Imaging department of TASH is among the most visited imaging units in the country. On average, at least one thousand CT-scan imaging are done every month (of which around 300 are abdominal CT scans).

**Study design and period**: An institution-based prospective cross-sectional study was conducted from 1 June 2020 – 31 August 2020. This period was chosen based on convenience considering the significant patient load reduction due to the COVID-19 pandemic.

**Source of data**: The source population was all adult patients referred from any unit of the TASH for diagnostic abdominal CT scans. All patients who were referred to the radiology department for abdominal CT examination for non-vascular indication during the specified period were taken as the study population

**Inclusion and exclusion criteria**: Clients who were 18 years old and above referred from all departments for abdominal CT for a non-major vascular indication like an abdominal aortic aneurysm or aortic stenosis were included in the study. Patients who were excluded from the study include those who:

- Were diagnosed with a chronic major vascular disease like an aortic aneurysm or aortic stenosis
- Had an abdominal mass compressing the aorta
- Were in acute cardiorespiratory distress
- Had depressed mentation
- Are of non-Ethiopian descendant

**Sampling procedure**: A convenience sampling method was used. All adult patients who came for abdominal CT scan evaluation for non-major vascular indications and who fulfil the inclusion criteria from June 1st to August 31st were included in the study.

**CT scanning techniques and measurement**: Abdomen CT was performed with 64 slice GE optima CT with a scan time of 0.6 seconds with a slice thickness of 5 mm. To minimize motion artifacts, patients were instructed to breathe at full inspiration and held his/her breath for 1s during real-time scanning. The original series of abdomen CTs which were taken with a slice thickness of 5 mm (volume scanning) were reconstructed with 1–3mm coronal and sagittal planes and sent to the PACS (picture archiving and communication system) workstation and images were reviewed by the investigators.

**Figure 1:**
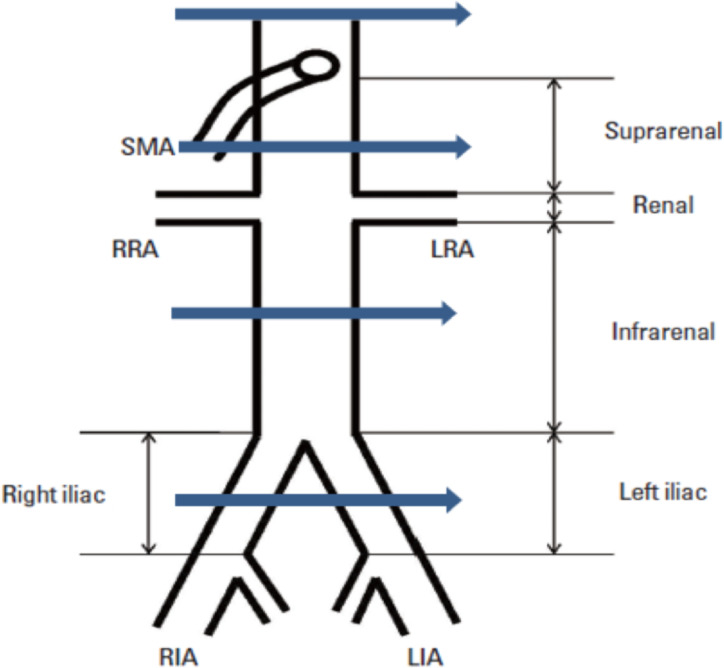
Levels of the abdominal aorta and common iliac arteries measurement (Source. – Modified from reference 12).

**Data collection tools and techniques**: Data was collected from the CT scan unit of the Radiology department of TASH starting from June 1^st^, 2020, up to August 30^th^, 2020. A structured questionnaire containing closed-ended questions specifically designed for the study was used for data collection. The questionnaire contains sociodemographic factors, clinical factors, and CT scan parameters. The demographic and clinical factors were directly interrogated from the patient after obtaining informed consent by two trained medical radiation technologists under close supervision and facilitation by the principal investigators before the procedure was performed. Every caution was taken for prevention of COVID-19 pandemic during interrogation. The CT scan findings were documented using a structured checklist later from the picture archiving and communication software by the investigators. Individual studies that fulfilled the inclusion criteria were reviewed from the PACS and the measurements of the abdominal aorta and bilateral common iliac arteries were taken from four distinct points: the aortic hiatus, midlevel between the SMA and renal artery, midlevel between the lower renal artery and aortic bifurcation, and midlevel between the aortic bifurcation and the origin of the internal iliac artery, as depicted in Figure 1 and documented in the questionnaire.

The dependent variables are anteroposterior and transverse diameters of the abdominal aorta at the aortic hiatus, supra, and infrarenal levels, and anteroposterior and transverse diameters of bilateral common iliac arteries. The Independent variables are the sociodemographic characteristics, weight, height, and BMI, Clinical profile of the patient including cardiac disease, diabetes mellitus, hypertension, substance use, and family history of diagnosed vascular disease.

**Operational definitions**

**Figure 2:**
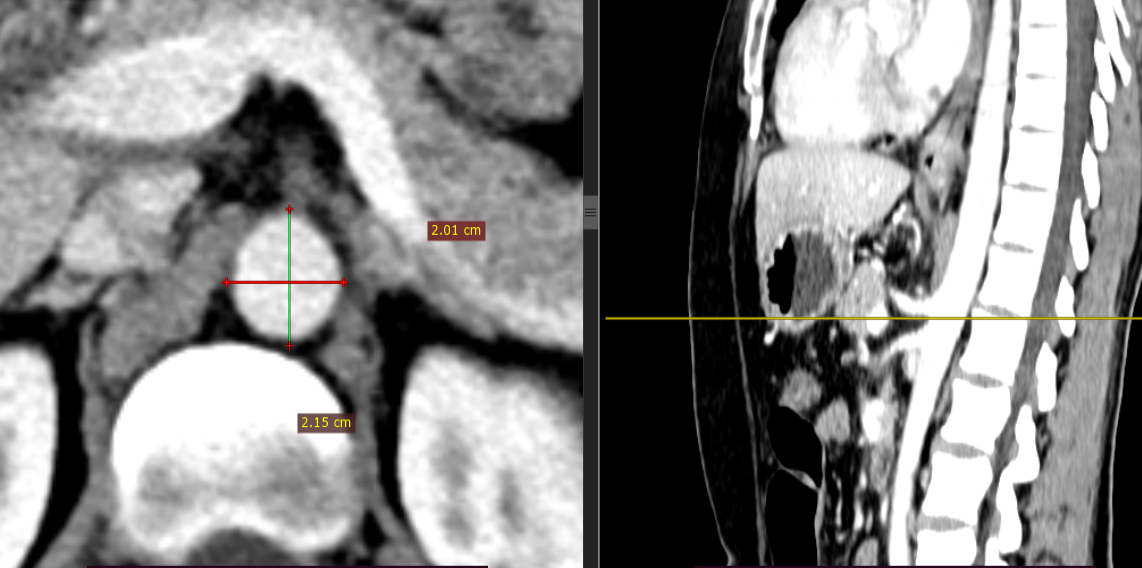
Axial image taken at T12 level (Aortic hiatus level) showing outer to outer CT measurement

**Aortic hiatus or T12 level aortic diameter**: stands for the maximal diameter of the aorta from the outer-to-outer layer at aortic hiatus or at T12 level as depicted in figure 2.

**Suprarenal aortic diameter**: stands for the maximal diameter of the aorta from the outer-to-outer layer at the mid-level between the superior mesenteric artery (SMA) and renal artery.

Infrarenal aortic diameter: refers to the maximal diameter of the aorta from the outer-to-outer layer at the mid-level between the lower renal artery and aortic bifurcation.

**Common iliac artery diameter**: refers to the maximal diameter of the common iliac artery from the outer-to-outer layer at the mid-level between the aortic bifurcation and the origin of the internal iliac artery.

**Data quality management**: The questionnaire used to collect data was prepared by the principal investigator in the English version. Brief training of the data collectors about the procedure of data collection was made before the actual data collection. Data collection was closely supervised and collected data was double-checked daily for consistency and completeness by the principal investigator.

**Data analysis**: Data entering, coding, and cleaning were performed using Epi-info version 7.2 and the analysis was done using SPSS version 22. The demographic & clinical characteristics of participants were computed by using simple descriptive statistics (mean, percentage, frequencies, and standard deviation). Pearson correlations were applied to calculate associations of aortic diameter with age, weight, height, and BMI at each level of aortic measurement. To analyze inter-group differences, student t-test and one-way ANOVA were used to compare the mean of aortic measures. A P-value of <0.05 and 95% confidence level was used as a difference of statistical significance.

**Ethical considerations**: Ethical clearance was obtained from the research and ethics committee of the department of radiology, AAU. All the study participants were informed about the purpose of the study and their right to refuse. Participants were also informed that refusal to participate will not affect subsequent medical care and then informed written consent was obtained. patient identifiers were not used in data collection and all personal information was kept confidential.

## Results

**Figure 3:**
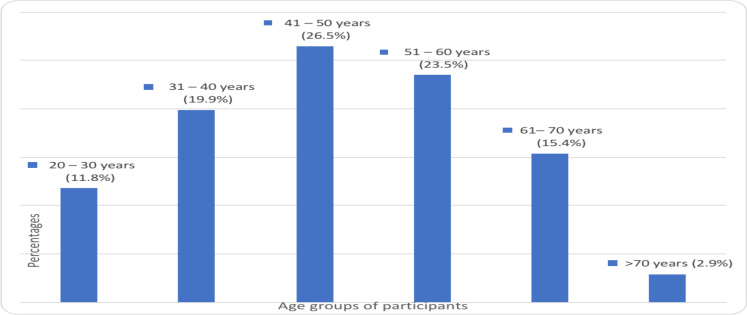
Frequency distribution by age category of patients for whom abdominal CT was done at TASH, 2020.

**Sociodemographic characteristics of the study participants**: A total of 136 patients were included in the study, of which, 81(59.6%) were females and 55(40.4%) were males. The age of participants ranged from 23 to 77 with a mean of 48.4±13.23 years. (Figure 3).

**Figure 4 (A & B):**
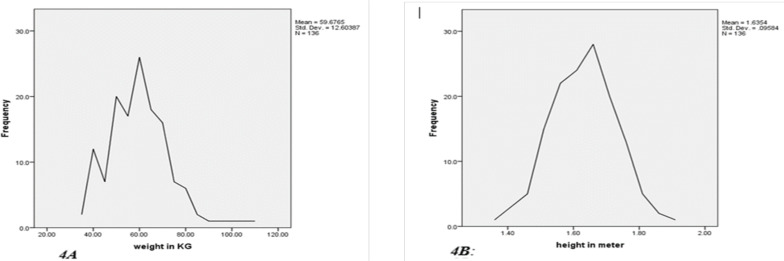
Height and Weight distributions of patients for whom abdominal CT was done at TASH and aortic and iliac measurements made, 2020.

The height distribution ranges from 1.36 to 1.90 meters with a mean height being 1.64±.10. The mean height of the study participants was 1.63±0.09m (Figure 4b), whereas the mean weight was 59kg±12.6kg (Figure 4a). The graphical weight distribution shows the minimum and maximum measurements of 35 and 110, respectively with a mean of 59.68±12.06 kg (Figure 4).

**Clinical findings**: In this study, four (2.9%), six (4.4%), eight (5.9%), and twelve (11%) of the patients had cardiac disease, diabetes mellitus, hypertension, and family history of vascular disease respectively. Fifteen (11%) of the study participants drink alcohol, while five (3.7%) were smokers (Table 1).

**Table 1:** Anteroposterior and transverse abdominal aortic and common iliac arterysizesat different anatomic levels on abdominal CT in TASH, 2020

Anatomic level	Dimensions	Male (n=55) Mean ± SD (cm)	Female (n=81) Mean± SD (cm)	Total (n = 136) Mean± SD (cm)
Aortic hiatus	Anteroposterior	2.27 ± 0.24	2.04 ± 0.22	2.13 ± 0.25
	Transverse	2.30 ± 0.25	2.03 ± 0.19	2.14 ± 0.25
	Average	2.28± 0.24	2.03± 0.20	2.13±0.25
Supra renal	Anteroposterior	2.01 ± 0.23	1.81 ± 0.23	1.90 ± 0.25
	Transverse	2.04 ±0.21	1.83 ±0.21	1.92 ± 0.23
	Average	2.03± 0.21	1.82± 0.21	1.91±0.23
Infra renal	Anteroposterior	1.75 ±0.16	1.53 ± 0.16	1.91 ±0.19
	Transverse	1.77 ±0.16	1.54 ±0.15	1.63 ±0.19
	Average	1.76± 0.16	1.54± 0.15	1.63± 0.19
Right common iliac	Anteroposterior	1.21 ± 0.15	1.02 ± 0.12	1.10 ± 0.16
	Transverse	1.23 ±0.15	1.05 ± 0.11	1.12 ±0.16
	Average	1.22± 0.15	1.04± 0.11	1.11± 0.16
Left common iliac	Anteroposterior	1.20 ± 0.16	1.02 ±0.12	1.09 ± 0.16
	Transverse	1.20 ± 0.15	1.03 ±0.11	1.10 ± 0.15
	Average	1.20± 0.15	1.03± 0.11	1.10± 0.16

**Aortic and common iliac artery size**: At the level of the aortic hiatus (T12), the average anteroposterior diameter was 2.13cm ±0.25cm and the transverse diameter was 2.14cm±0.25cm (Table 2). At the level midway between the aortic bifurcation and the termination of the common iliac arteries, the mean anteroposterior and transverse diameters of the left common iliac artery were 1.09cm and 1.1cm respectively (Table 1).

**Table 2:** Correlation of aortic and common iliac arteries diameter with age, weight, height, BSA, and BMI among adults who undergone Abdominal CT examination in TASH, 2020

Anatomic measurement		Age	weight in kg	height in meter	BMI	BSA
Aortic diameter at Aortic Hiatus level	Pearson Correlation	.446**	.372**	.394**	.143	.427
	Sig. (2-tailed)	.000	.000	.000	.097	.000
	N	136	136	136	136	136
Aortic diameter at supra renal level	Pearson Correlation	.353**	.409**	.423**	.164	.467
	Sig. (2-tailed)	.000	.000	.000	.057	.000
	N	136	136	.136	136	136
Aortic diameter at Infra renal level	Pearson Correlation	.360**	.536**	.519**	.236**	.600
	Sig. (2-tailed)	.000	.000	.000	.006	.000
	N	136	136	136	136	136
Right common iliac artery	Pearson Correlation	.305**	.582**	.532**	.268**	.648
	Sig. (2-tailed)	.000	.000	.000	.002	.000
	N	136	136	136	136	136
Left common iliac artery	Pearson Correlation	.258**	.613**	.526**	.306**	.678
	Sig. (2-tailed)	.002	.000	.000	.000	.000
	N	136	136	136	136	136
Age	Pearson Correlation	1	.043	-.002	.052	.035
	Sig. (2-tailed)		.622	.978	.544	.687
	N	136	136	136	136	136
weight in KG	Pearson Correlation	.043	1	.365**	.814**	.973
	Sig. (2-tailed)	.622		.000	.000	.000
	N	136	136	136	136	136
height in meter	Pearson Correlation	-.002	.365**	1	-.231**	.564
	Sig. (2-tailed)	.978	.000		.007	.000
	N	136	136	136	136	136
BMI	Pearson Correlation	.052	.814**	-.231**	1	.666
	Sig. (2-tailed)	.544	.000	.007		.000
	N	136	136	136	136	136
BSA	Pearson Correlation	.687	.973	.564	.666	1
	Sig. (2-tailed)	.035	.000	.000	.000	
	N	136	136	136	136	136

** Correlation is significant at the 0.01 level (2-tailed)

**Factors associated with aortic and common iliac arteries size**: The mean anteroposterior and transverse aortic and common iliac arteries diameter had a significant positive correlation) with age, weight, height, BSA, and BMI (p<0.05 as shown in Table 2.

The result of the independent samples t-test showed the presence of a statistically significant difference between the sex and diameters. The mean aortic and common iliac diameters measured at all anatomical levels were larger in males than in females (Table 3).

**Table 3:** Summary of the independent samples t-test between aortic and common iliac arteries diameter and sex of patients who had Abdominal Computed Tomography imaging in TASH, 2020

		t-test for Equality of Means						
		
Independent Sample Test		T	Df	Sig. (2-tailed)	Mean Difference	Std. Error Difference	95% Confidence Interval of the Difference	
							Lower	Upper
Aortic Hiatus	Equal variances assumed	6.670	134	.000	.24945	.03740	.17548	.32342
	Equal variances not assumed	6.440	101.555	.000	.24945	.03873	.17262	.32629
supra renal	Equal variances assumed	5.640	134	.000	.20674	.03666	.13424	.27924
	Equal variances not assumed	5.645	116.443	.000	.20674	.03662	.13420	.27928
Infra renal	Equal variances assumed	8.359	134	.000	.22341	.02673	.17055	.27628
	Equal variances not assumed	8.296	113.038	.000	.22341	.02693	.17006	.27676
Right iliac	Equal variances assumed	8.521	134	.000	.18756	.02201	.14403	.23110
	Equal variances not assumed	7.983	89.595	.000	.18756	.02349	.14088	.23424
left Iliac	Equal variances assumed	7.691	134	.000	.17393	.02262	.12920	.21866
	Equal variances not assumed	7.244	91.634	.000	.17393	.02401	.12625	.22162

Age was found to have a statistically significant association with aortic (at all levels) and right common iliac diameter with increasing diameters being detected as the age of the participant increased. The left common iliac artery did not show any statistically significant association with age (Table 4).

**Table 4:** Mean diameter of the abdominal aorta by age category at different anatomic levels on Abdominal CT scan in Tikur Anbessa Specialized Hospital, 2020 (N=136)

Age in years	Number	Aortic hiatus level Mean ± SD (cm)	Suprarenal level Mean ± SD (cm)	Infra renal level Mean ± SD (cm)	Mid Right common iliac artery Mean ± SD (cm)	Left common iliac artery Mean ± SD (cm)
20– 30	16	1.96±.22	1.74±.19	1.53±.16	1.04±.13	1.04±.13
31– 40	27	2.03±.24	1.85±.218	1.54±.20	1.07±.13	1.06±.14
41 – 50	36	2.11±.23	1.89±.23	1.62±.16	1.09±.16	1.08±.16
51 – 60	32	2.21±.22	1.98±.24	1.69±.19	1.16±.16	1.14±.17
61–70	21	2.29±.22	2.00±.20	1.6±.18	1.14±.15	1.12±.13
>70	4	2.26±.25	2.03±.13	1.74±.19	1.27±.15	1.24±.19

## Discussion

Our study showed the mean diameter of the abdominal aorta at both suprarenal and infrarenal levels and bilateral common iliac arteries. The suprarenal abdominal aorta anteroposterior mean diameter was 2.01 ± 0.23 cm in males and 1.81 ± 0.23 cm in females while the transverse mean diameter was 2.05 ±0.21 cm in males and 1.83 ±0.21 in females. The infrarenal abdominal aorta anteroposterior diameter was 1.75cm ±0.16cm in males and 1.53cm ± 0.16cm in females while the average transverse diameter was 1.77 cm±0.16cm in males and 1.54cm ±0.15cm in females. This study has found that aortic size measurements done in transverse planes are slightly higher than Antero-Posterior diameters.

Our study has also shown that gender difference exists in the aortic dimension at all levels which most other studies in Africa and Asian countries have established with the mean aortic size in males being larger than females by about 2mm(10–13). There are also variations in the mean aortic diameter at different levels with body surface area and BMI, as found in ours and in other studies. (14, 15).

The progressive increment in the size of the abdominal aorta with age in both males and females in our study is also in line with most other works of literature (11, 12, 15). This shows that knowledge of a local reference range for abdominal aortic size is necessary as it enables to identify and define different vascular abnormalities in the population. Establishing a normal reference value will also be helpful in the selection of the appropriate stent size for vascular interventional procedures (16).

Even though an association between smoking and aortic size has been observed in other studies (19), our study didn't show such associations possibly due to small sample size and a small number of smokers among the study participants.

Common iliac dimensions in our study showed slight variation with other similar studies done in Korea and Norway though though studies used ultrasound to determine iliac artery dimensions(15, 20). which might explain the variation.

With the objective of early detection and management of abdominal aortic aneurysms, many countries have introduced screening programs in order to minimize morbidity and mortality associated with aortic and iliac arterial pathologies. In this regard, this study will be an important step before introducing such programs in our country as it provides normal reference data on the abdominal aorta and common iliac arteries in both men and women.

Evaluation of the abdominal aorta and common iliac artery diameters will also guide health policymakers and clinicians to design strategic programs on inculcating imaging-assisted cardiovascular screening protocol in candidate populations. Finally, the findings obtained from this study will serve as a baseline for future research.

As this is a study from a single institution, it may not represent the actual dimensions of the abdominal aorta and iliac arteries of the general population. The Small sample size is also an additional limitation of this study. In addition, risk factors that showed a significant correlation with aortic and iliac diameters were not quantified. Despite the limitations, it serves as a baseline for further large-scale studies. Generally, our study has shown that the diameter of the abdominal aorta at different levels varies and it also showed similarities with other regional Studies having statistically significant differences in size between males and females with the former having a larger size at all levels

Recommendation: The authors recommend a multicentre large-scale study to address geographic and ethnic variations.

## Figures and Tables

**Table 5:** Distribution of Abdominal aortic and iliac diameters by BMI at different anatomic levels in TASH, 2020 N=136

BMI	Number	Aortic hiatus Mean ± SD(cm)	Suprarenal Mean ± SD(cm)	Infra renal Mean ± SD(cm)	Right Iliac Mean ± SD(cm)	Left Iliac Mean ± SD(cm)
< 18.5	25	2.08 ± 0.24	1.81 ± 0.22	1.55 ±0.16	1.02 ± 0.14	0.98 ±0.11
18.5 – 24.9	77	2.13 ± 0.26	1.90 ±0.23	1.61 ±0.19	1.12 ±0.14	1.10 ±0.15
25 – 29.9	25	2.16 ± 0.21	1.96 ± 0.20	1.67 ± 0.16	1.13 ± 0.13	1.13 ±0.12
30 – 34.9	7	2.15 ± 0.26	1.98 ± 0.27	1.74 ±0.24	1.19 ± 0.25	1.17 ±0.214
≥35	1	2.22	1.83	1.67	1.09	1.005
